# Modeling of seizure transitions with ion concentration dynamics

**DOI:** 10.1186/1471-2202-16-S1-P242

**Published:** 2015-12-18

**Authors:** Damiano Gentiletti, Marco De Curtis, Vadym Gnatkovski, Piotr Suffczynski

**Affiliations:** 1 Department of Experimental Physics, University of Warsaw, Warsaw, Poland; 2 Fondazione Istituto Neurologico Carlo Besta, Milan, Italy

## 

Traditionally it is considered that neuronal synchronization in epilepsy is caused by a chain reaction of synaptic excitation. However, it has been shown that synaptic transmission is not necessary for epileptiform synchronization [1]. In order to investigate the respective roles of synaptic and non-synaptic neuronal coupling in seizure transitions, we developed a computational model of hippocampal network, involving extracellular space, realistic dynamics of Na^+^, K^+ ^and Cl^- ^ions, the glial uptake and diffusion mechanism. We show that network behavior under synaptic coupling conditions may be quite different from the neurons' activities when specific non-synaptic components are included. In particular, we show that in the extended model, strong discharge of inhibitory interneurons may result in long lasting accumulation of extracellular K^+^, which sustains depolarization of principal cells and causes their pathological discharges. This effect is not present in a reduced, purely synaptic network. These results confirm the experimental hypothesis that increase of inhibitory interneurons firing may lead to increased firing in the pyramidal cells through accumulation of extracellular potassium [2]. The model also shows that all potassium clearance mechanisms (glial uptake, sodium-potassium pump, potassium diffusion) are critically important to reproduce the experimental findings. This means that computational modeling of seizure activity without ion dynamics may lead to unrealistic results.
